# Status of the enigmatic Oriental genus *Rhithrogeniella* Ulmer, 1939 (Ephemeroptera, Heptageniidae)

**DOI:** 10.3897/zookeys.429.8116

**Published:** 2014-07-29

**Authors:** Michel Sartori

**Affiliations:** 1 Zoologisches Museum und Biozentrum Grindel, Martin-Luther-King-Platz 3, D-20146 Hamburg, Germany; 2 Museum of Zoology, Palais de Rumine, Place Riponne 6, CH-1005 Lausanne, Switzerland

**Keywords:** *Rhithrogeniella ornata*, *Rhithrogeniella tonkinensis*, *Nixe*, *Paracinygmula*, new combinations, Sumatra, Java

## Abstract

Based on historic collections and new material from Sumatra and Java, the species *Rhithrogeniella ornata* Ulmer, 1939, type species of the genus *Rhithrogeniella*, is reinvestigated. The nymph is described for the first time and is closely related to the continental Southeast Asian species *Rhithrogeniella tonkinensis* Soldán and Braasch, 1986. *Rhithrogeniella* belongs to the subfamily Ecdyonurinae, and is related to the genera *Nixe* Flowers, 1980 and/or *Paracinygmula* Bajkova, 1975 based on characters of the nymphal stage. Species described from Taiwan in the genus *Nixe* are transferred to the genus *Rhithrogeniella*: *Rh. littoralis* (Kang and Yang, 1994) **comb. n.**, *Rh. mitifica* (Kang and Yang, 1994) **comb. n.** and *Rh. obscura* (Kang and Yang, 1994) **comb. n.**

## Introduction

Ulmer (1939) established the genus *Rhithrogeniella* to accommodate the species *Rhithrogeniella ornata* Ulmer, 1939 known from imagos and subimagos from Java and Sumatra. According to Ulmer (1939), the genus was characterized by genitalia resembling those of *Rhithrogena*, with two simple lobes lacking spines or titillators. On the other hand, the new genus differed from *Rhithrogena* in the tarsal proportions on fore- and hind legs. Interestingly, in the key to the genera (Ulmer 1939), *Rhithrogeniella* is keyed with the genus *Afronurus* Lestage, 1924 from which it differs by the tarsal composition of the foreleg. During the following years, nothing substantial was added to the knowledge of the genus, and keys which included *Rhithrogeniella* were based on Ulmer’s description and drawings (Tomka and Zurwerra 1985; Tshernova 1974).

Major advancement was made by Soldán and Braasch (1986) who described a new species from Vietnam *(Rh. tonkinensis)* based on subimagos, together with the first description of the nymphal stage. This nymph exhibits typical Ecdyonurinae morphology, with scattered setae on the maxillae. The genus can be distinguished from all relatives by the peculiar structure of the cerci and terminal filament which possess a row of stout setae in the proximal part and bunches of long and thin setae in the medial and distal parts. Later, *Rhithrogeniella tonkinensis* was reported from Thailand and the male imago described (Braasch 1990). According to figures of Braasch (1990), the genitalia bear median titillators, the penis lobes are much more rounded than in *Rhithrogeniella ornata*, and the styliger fig is of an unusual shape with two triangular sublateral processes. In their revision of worldwide Heptageniidae, Wang and McCafferty (2004) proposed several nomenclatorial changes; in particular, they combined *Rhithrogeniella ornata* with the genus *Rhithrogena*, hence placing *Rhithrogeniella* in synonymy with *Rhithrogena*, and they assigned *Rhithrogeniella tonkinensis* to the genus *Ecdyonurus (E. tonkinensis)* based on subimaginal, larval and egg morphology. This account was never discussed later on and Braasch and Boonsoong (2010) mentioned the presence of *Rhithrogeniella ornata* in West Malaysia based on male subimagos, and Boonsoong and Braasch (2013) listed *Rhithrogeniella tonkinensis* in the Heptageniidae fauna of Thailand.

Two questions need to be resolved. Are Wang and McCafferty (2004) correct in synonymizing *Rhithrogeniella* with *Rhithrogena* (subfamily Rhithrogeninae), and assigning *Rhithrogeniella tonkinensis* to the genus *Ecdyonurus* (subfamily Ecdyonurinae)? Is the association between unreared nymphs and subimagos of *Rhithrogeniella tonkinensis* accurate or not?

The type material of *Rhithrogeniella ornata*, deposited in the collection of the Zoological Museum of Hamburg University, Germany (ZMH) has been reinvestigated together with new material from Sumatra. It is now possible to provide the first description of the nymph of *Rhithrogeniella ornata*.

## Material and methods

Material studied here is deposited in the following institutions:

Zoologisches Museum und Biozentrum Grindel, Hamburg, Germany [ZMH]

Musée cantonal de zoologie, Lausanne, Switzerland [MZL]

Lembaga Ilmu Pengetahuan Indonesia (Indonesian Institute of Sciences), Museum of Zoology, Bogor, Indonesia [LIPI] (Bogor was formerly known as Buitenzorg)

Drawings were made with the help of a camera lucida taken from stereomicroscope Leica DM 750 and pictures from microscope Zeiss Axioscop 2 or Visionary Digital Passport II. Final digital drawings were performed on Adobe Illustrator CS6. For scanning electronic microscope (SEM) pictures, the eggs were dehydrated, carbon coated, and observed under a LEO 1525 at 5.00kV; maxillae were dehydrated, critical point dried, and then platinum coated, and observed under a FEI Quanta 250 at 5.00kV. Final figs were assembled in Adobe Photoshop CS6.

Nymphs and adults were associated with the help of the egg structure (Fig. 5).

## Results

### Rhithrogeniella ornata

Taxon classificationAnimaliaEphemeropteraHeptageniidae

Ulmer, 1939

Rhithrogeniella ornata Ulmer, 1939, male, female imagos and subimagos
Rhithrogena ornata Wang & McCafferty, 2004

#### Material.

One male holotype, one female allotype: Indonesia, Java, Buitenzorg, VII 1932, Dr. Lieftinck leg. [ZMH]

Paratypes: 4 female subimagos, 1 male subimago: Indonesia, Java, Buitenzorg, Bellevue, caught at light, VII.1929, Prof. Thienemann leg. [ZMH]; 4 female imagos, 2 male subimagos: Indonesia, Sumatra, Padang, VII 1925, Prof. Fulmek leg. [ZMH]; 1 male subimago: Indonesia, Sumatra, Pangkalang, Kota baru, X 1925, Prof. Fulmek leg. [ZMH]

All specimens in ethanol, except fore- and hind legs, fore- and hind wings of the male subimago from Buitenzorg mounted on slide in Canada balsam.

Other material: 5 nymphs: Indonesia, Sumatra Barat, Sawahlunto, stream, 275m, 00°41.33'S, 100°46.72'E, (UN5), 10.XI.2011, M. Balke leg. [ZML]; 26 nymphs, of which two entirely mounted on microscopic slides: Indonesia, Sumatra Barat, Talawi, Ombilin River, 277m, 00°34.15'S, 100°43.54'E, (UN4), 8.XI.2011, M. Balke leg. [ZMH, MZL, LIPI]

#### Complementary description of the male imago (holotype).

Specimen completely faded; for color patterns see Ulmer (1939).

Mesonotum with transverse suture; medial depression of furcasternum sub parallel anteriorly.

Foreleg with tarsi sub equal in length to the tibia, which is 1.25x longer than the femur. Tarsal composition: 2>3>4>5>1.

**Figures 1–4.**
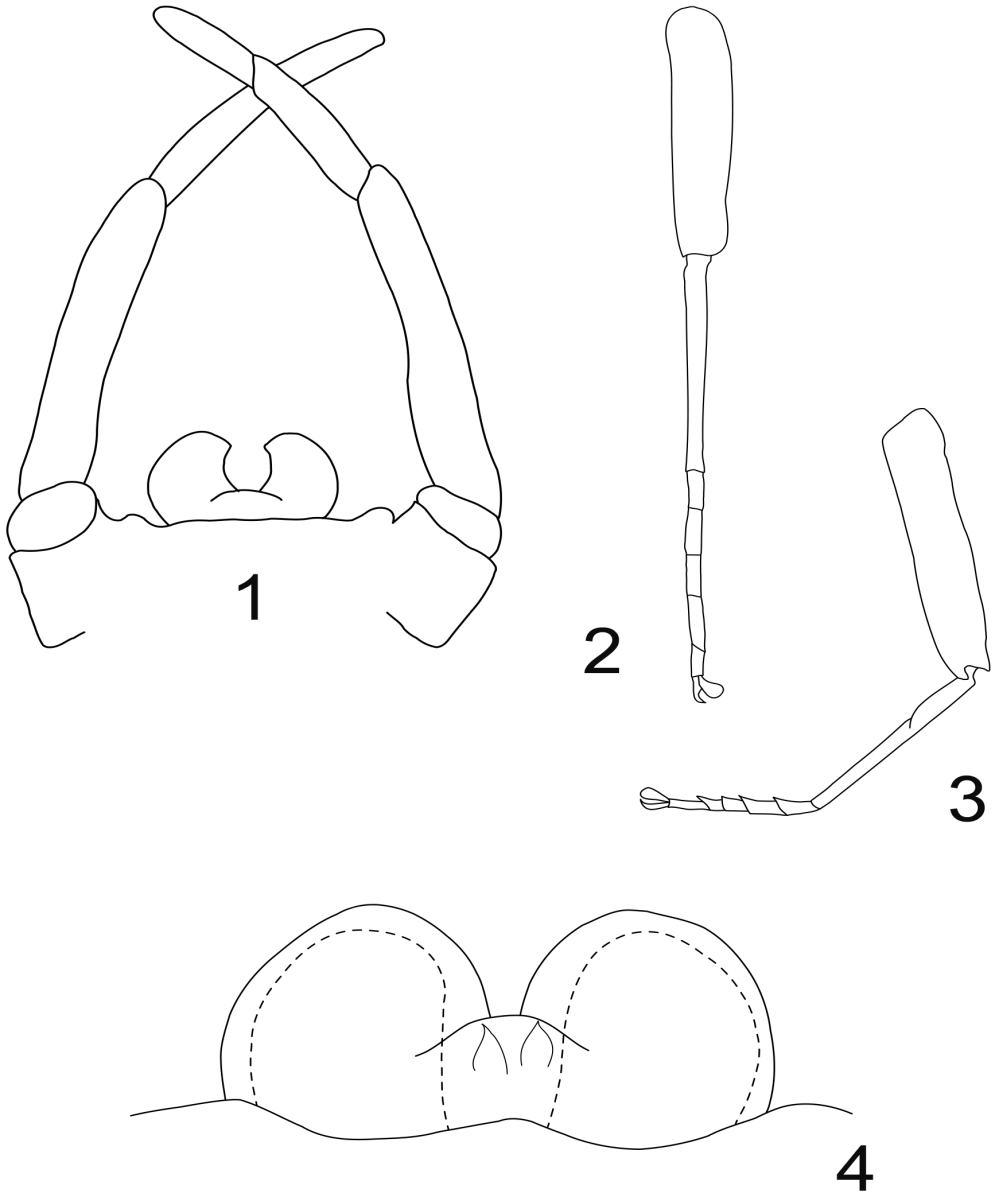
*Rhithrogeniella ornata* Ulmer, 1939. **1** Genitalia of the male imago (holotype) in ventral view **2** Foreleg of a male subimago (paratype) **3** Hindleg of a male subimago (paratype) **4** Penis lobes of a male subimago (paratype): plain line, cuticular structures of the subimago; dotted line, outline of the imago penis lobes.

Genitalia (Fig. 1): margin of the styliger fig straight to slightly convex, with two small sub-lateral rounded processes; last gonopod segment ca 0.7× the length of the previous, both together ca 0.75× the length of the antepenultimate. Penis constituted of two kidney-shape lobes, separated by a “U” incision, i.e. the inner margin of each lobe is concave and slightly hooked near the apex. No lateral or median titillators, no apical spines visible.

#### Complementary description of the male subimago.

Fore leg (Fig. 2) with femur ca 1.15x the length of tibia, which is subequal in length to tarsi. Tarsal composition 4≥2>3≥1>5.

Hind leg (Fig. 3) with femur ca 1.35x the length of tibia, which is ca 1.45× the length of tarsi. Tarsal composition 1=2=5>3≥4.

Genitalia (Fig. 4) with penis lobes rounded, ellipsoid, without any spine or titillators; in median position, a pair of membranous processes ending with a spine like sclerotization present in ventral view.

#### Complementary description of the female imago (allotype).

Thoracic structures similar to the male.

**Figure 5.**
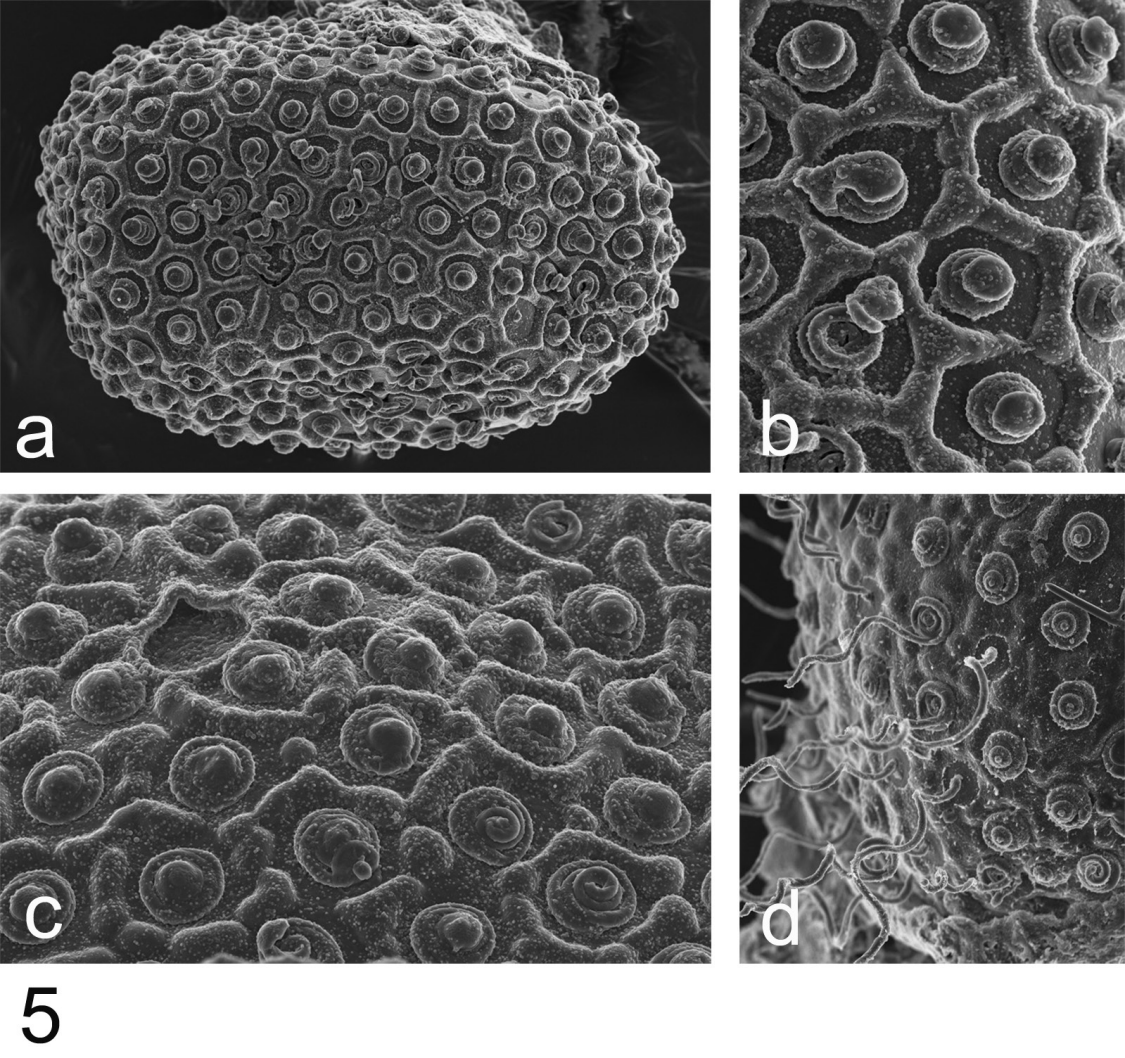
*Rhithrogeniella ornata* Ulmer, 1939, SEM pictures of egg structures. **5a** Egg extracted from a female subimago paratype from Padang, Sumatra **5b** Details of the chorionic structure of a female nymph from Ombilin River, Sumatra **5c** Details of the chorionic structure and micropyle of a female subimago paratype from Buitenzorg [Bogor], Java **5d** chorionic surface of the female allotype from Buitenzorg [Bogor], Java.

Eggs (Fig. 5): ovoid, ca 130 µm × 90 µm; chorion regularly covered with hexagonal mesh ridges, with KCT in-between, not larger at poles; micropyle rounded to slightly oval in equatorial area.

#### First description of the nymph.

Size: Body length: up to 5.2 mm and 5.6 mm for male and female respectively; cerci and terminal filament subequal and ca ¾ the length of the body.

**Figures 6–7.**
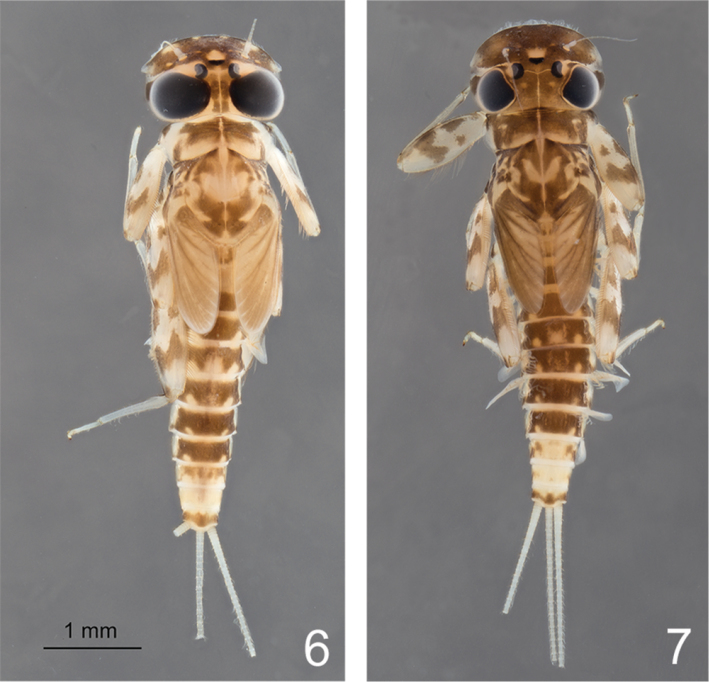
*Rhithrogeniella ornata* Ulmer, 1939. **6** Male nymph **7** Female nymph with slight color variations.

Coloration similar to Figs 6 and 7.

**Figures 8–11.**
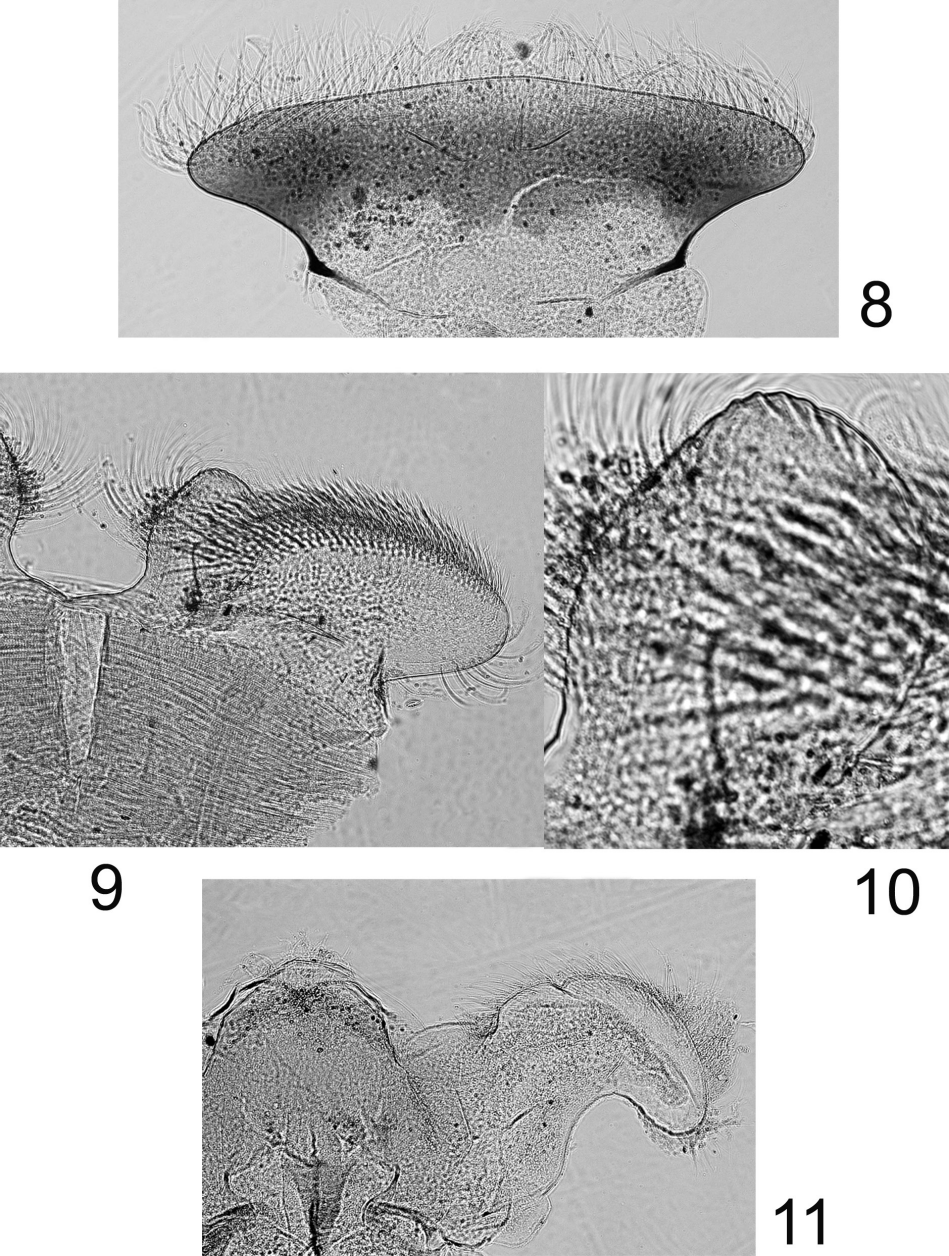
*Rhithrogeniella ornata* Ulmer, 1939, nymphal mouthparts. **8** Labrum in dorsal view **9** Left glossae and paraglossae of the labium **10** Detail of the glossae from **9 11** Hypopharynx, ventral view lingua and left superlingua.

**Figures 12–14.**
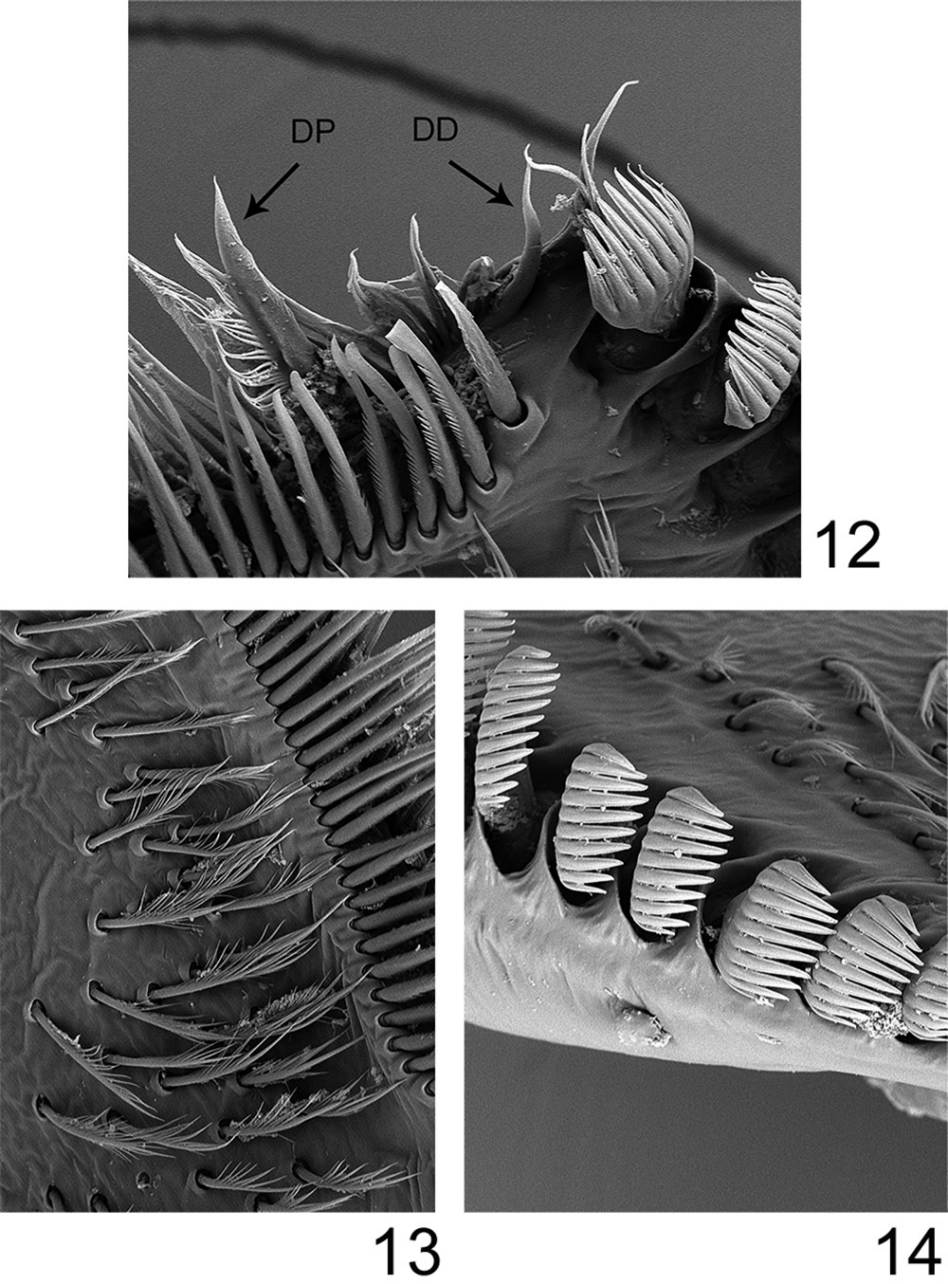
*Rhithrogeniella ornata* Ulmer, 1939, SEM pictures of the maxilla. **12** Dentisetae (DP: proximal dentiseta, DD: distal dentiseta) **13** Fimbriate setae on the ventral surface **14** Comb-shape setae on the crown of the galea-lacinia.

Labrum (Fig. 8) moderately expended laterally, ca 2.6× wider than long; lateral margins regularly rounded; no anteromedian emargination; dorsal face covered with long and thin setae anteriorly; ventral face with shorter and stout setae along the anterior margin. Mandibles covered with numerous long and thin setae on the outer margin; right mandible with outer incisor saw-like, inner one with a trifid apex with 2–3 pectinate setae below it, and 2–3 long and simple setae below the mola; left mandible with outer incisor saw-like, inner one with a bifid apex with 3–4 pectinate setae below it, and 3–4 long and simple setae below the mola. Maxillary palp three-segmented; first segment covered with thin setae on inner and outer margin; second segment with thin setae on the outer margin; third segment slightly pointed, only with long and thin setae. Maxillae with fimbriate scattered setae on the ventral surface (Fig. 13): 13–14 comb-shape setae on the crown of the galea, median ones with 10–11 teeth (Fig. 14); proximal dentiseta bifid, outer margin feathered; distal dentiseta simple, entire and unbranched (Fig. 12). Labium (Fig. 9) with glossae rhomboid, inner margin covered with long and thin setae, apex characteristic with scale-like margin (Fig. 10); paraglossae moderately expended laterally. Hypopharynx (Fig. 11) with rhomboid lingua bearing a tuft of short and thin setae at apex; superlinguae well developed and expended laterally with rounded apex and setae on the outer margin extended beyond the apex.

**Figures 15–19.**
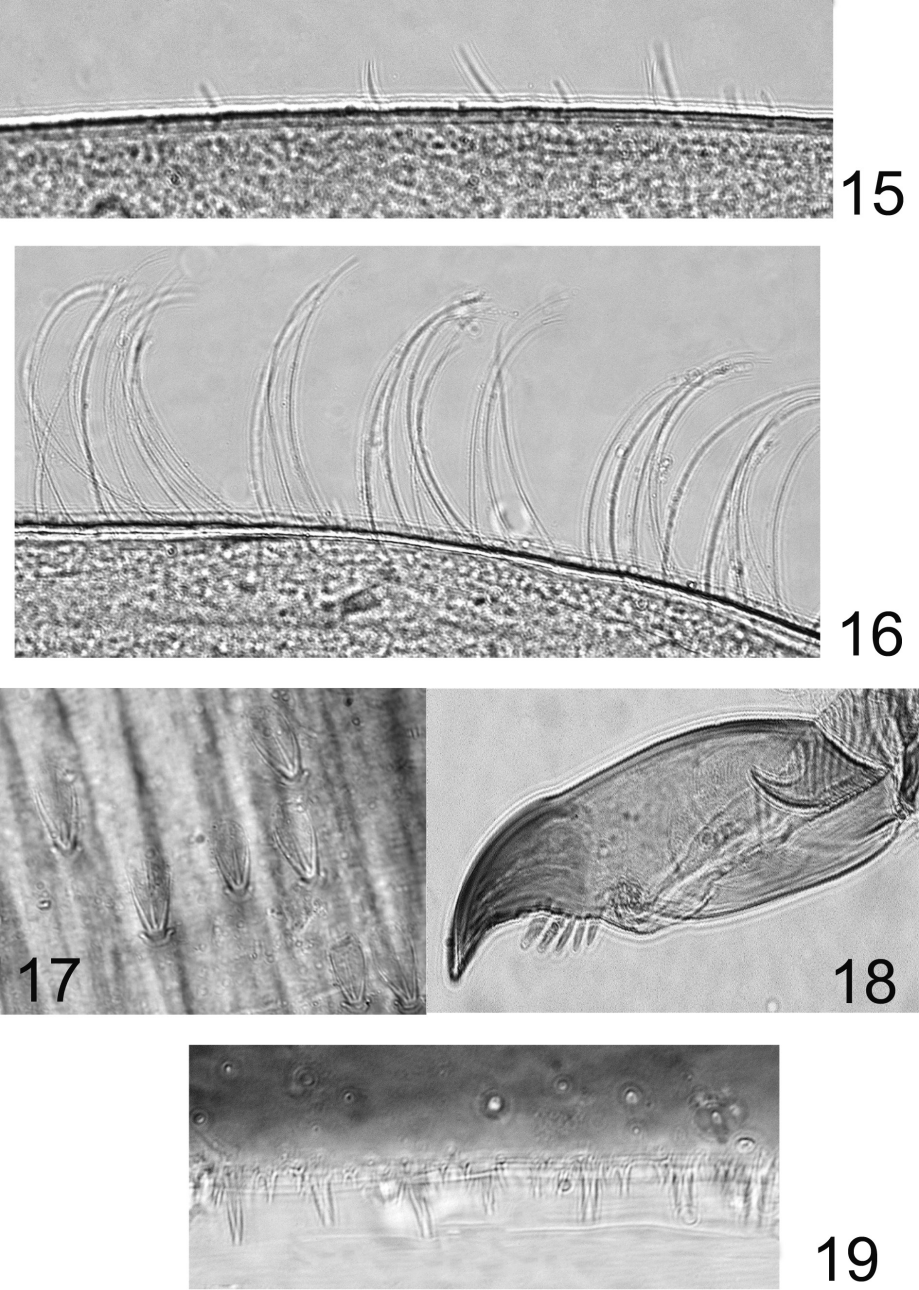
*Rhithrogeniella ornata* Ulmer, 1939. **15** Outer margin of the fore tibia **16** Outer margin of the hind tibia **17** Bristles on the dorsal surface of hind femur **18** Tarsal claw **19** Posterior margin of tergite V.

Pronotum moderately expended laterally. Foreleg with femur ca 2.6× longer than wide; outer margin covered with long and stout setae, becoming thinner near the apex; inner margin with only few spine-like setae on the distal third. Outer margin of tibia with very few thin and short setae (Fig. 15), inner margin with few spine-like setae in the middle; tarsi with only a few spine-like setae in the middle of the inner margin. Hind leg similar, except the spine-like setae on inner margin of the femur present on the whole margin; outer margin of tibia with a row of long and thin setae (Fig. 16) and inner margin with more numerous spine-like setae. Middle leg similar to hind leg, except spine-like setae on the inner margin of the femur only present on the distal half. Bristles on the upper face of femora variable in length, always with divergent margins and rounded apically (Fig. 17). Tarsal claw moderately hooked, bearing 4–6 teeth (Fig. 18). No supracoxal spurs present.

**Figures 20–23.**
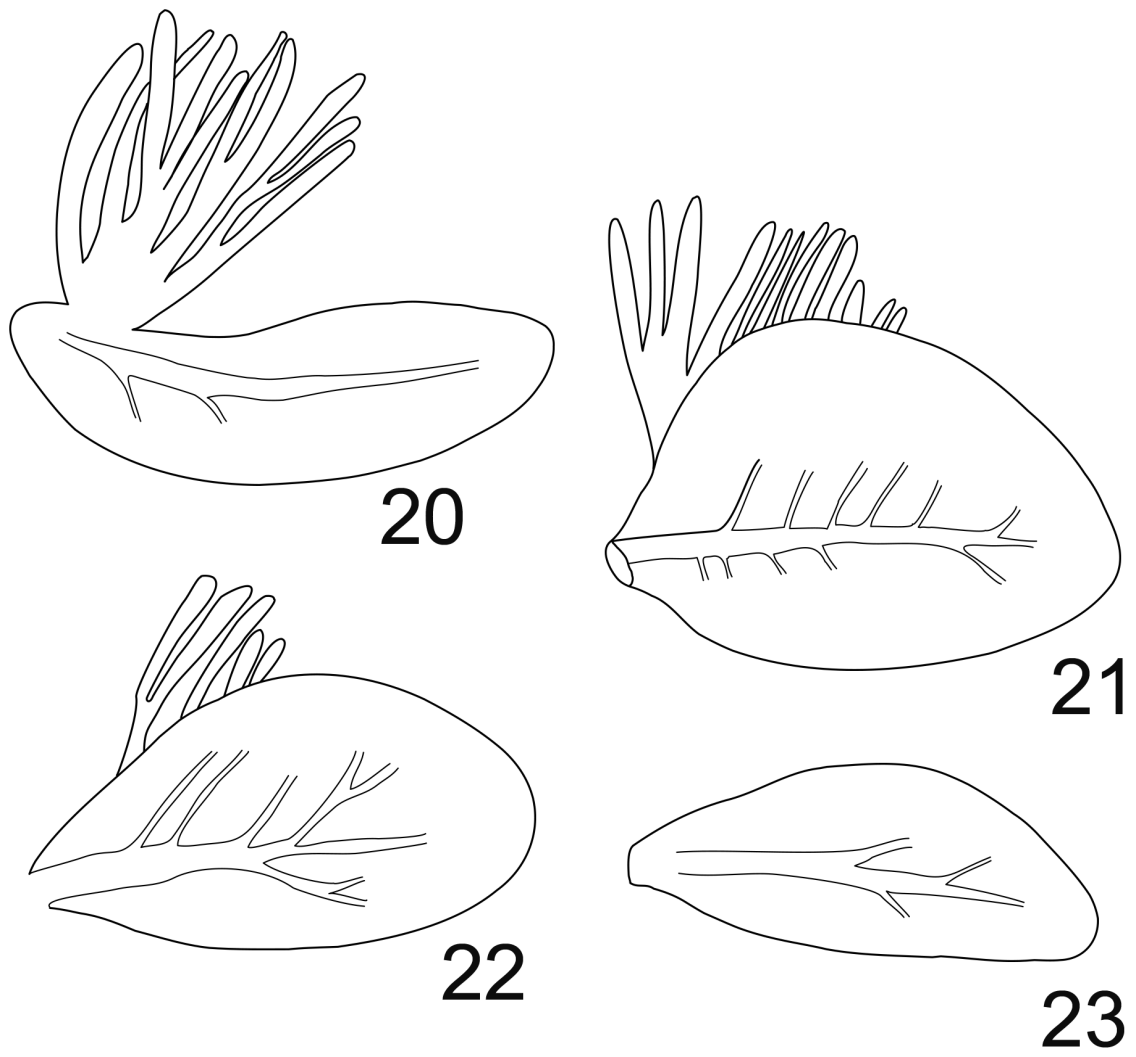
*Rhithrogeniella ornata* Ulmer, 1939. **20** Gill I **21** Gill IV **22** Gill VI **23** Gill VII.

**Figures 24–25.**
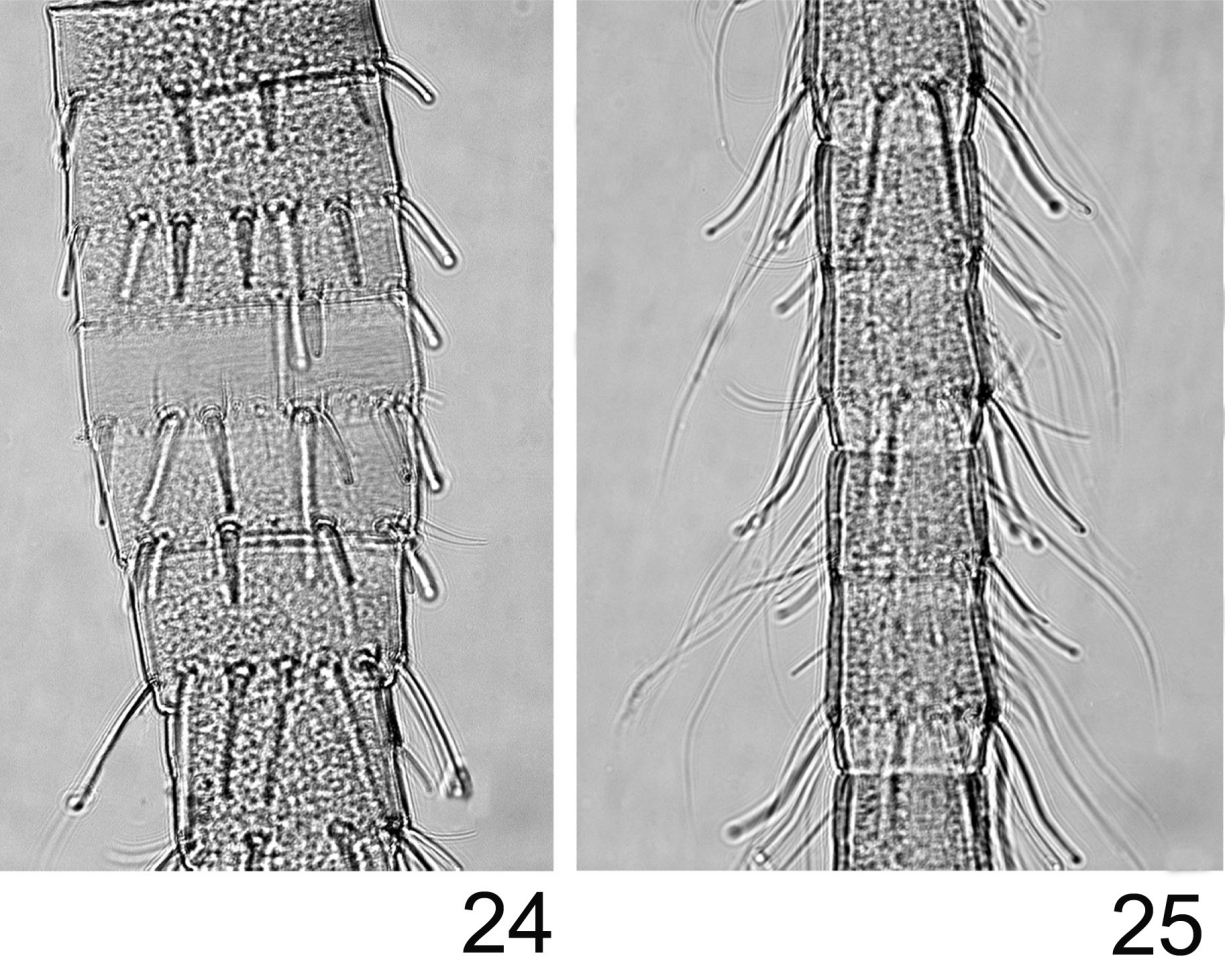
*Rhithrogeniella ornata* Ulmer, 1939. **24** Proximal part of the terminal filament **25** Median part of the terminal filament.

Abdomen with posterolateral extensions weakly developed, visible only on segments V–VIII. Gills present on abdominal segments I–VII. Gill I banana-shape (Fig. 20), with fibrillar part well developed, gill IV ca 1.5× longer than wide, strongly asymmetrical (Fig. 21), gill VI with well-developed fibrillar part, more elongated and slightly asymmetrical (Fig. 22), gill VII ca 2.5× longer than wide, without fibrillar part and slightly asymmetrical (Fig. 23). Posterior margin of abdominal terga with weakly developed spines of different size and shape (Fig. 19). Cerci and terminal filament with long and stout setae in whorls on the proximal part (Fig. 24), together with long and thin setae in the median and distal part (Fig. 25).

#### Sequence data.

One specimen has been used for the study by Vuataz et al. (2013) under the name “Heptageniidae 1” in figures and “Heptageniidae sp. 1” in table S1, with one mitochondrial (CO1) and two nuclear genes (H3, wg) sequenced. Access numbers in GenBank are for CO1: HF536605, for wg: HF536598, for H3: HF536591.

## Discussion

The genitalia of the male imago differ slightly from those described by Ulmer (1939, page 577, fig. 169), being wider and less cylindrical than illustrated. The presence of a transverse suture on the mesonotum together with the shape of the depression of the furcasternum (not narrowed anteriorly) indicates that *Rhithrogeniella ornata* cannot be a member of Rhithrogeninae; thus, it is not a synonym of *Rhithrogena* as suggested by Wang and McCafferty (2004). Moreover, the presence of scattered setae on the ventral side of the maxilla is a character only found among members of Ecdyonurinae, as suggested already by Soldán and Braasch (1986). When using the key of Webb and McCafferty (2008), *Rhithrogeniella* will key to the genus *Afronurus* Lestage, 1924 for the adults and to the genus *Nixe* Flowers, 1980 for the nymphs. Nearctic workers consider the genus *Nixe* as valid (Flowers 1986; McCafferty 2004; Wang and McCafferty 2004; Webb and McCafferty 2008), but European authors think that its concept is similar to *Paracinygmula* Bajkova, 1975 (Jacob et al. 1996). The Holarctic species *joernensis* (Bengtsson, 1909) is therefore treated either as *Paracinygmula joernensis* (Bauernfeind and Soldán 2012) or as *Nixe joernensis* (Kjaerstad et al. 2012). Nymphs of *Rhithrogeniella* share some characters with this concept, including the presence of swimming setae on cerci and terminal filament, and the chorionic structures of the eggs (Flowers 1980). Nymphs of *Nixe/Paracinygmula* however present gills with a weakly developed fibrillose part, either absent or reduced to a single filament in gill VI, which is not the case in *Rhithrogeniella* (Fig. 22 and Soldán and Braasch, 1986, fig. 4). Contrary to *Nixe/Paracinygmula*, the male genitalia have a very different shape and lack well developed median titillators as well as basal sclerite spines.

Three species of *Nixe* known only from the nymphal stage are reported from Taiwan (Kang and Yang 1994). Bauernfeind and Soldán (2012) transferred them to the genus *Paracinygmula* without new data, because they considered *Nixe* as a subjective junior synonym of *Paracinygmula*. Examination of paratypes of these species, deposited in the collections of MZL, revealed that they perfectly match the concept of *Rhithrogeniella* developed here, and therefore the following new combinations are proposed:

*Rhithrogeniella littoralis* (Kang and Yang 1994) comb. n. (= *Nixe (Nixe) littoralis* Kang and Yang 1994 =*Paracinygmula littoralis* Bauernfeind and Soldán 2012;

*Rhithrogeniella mitifica* (Kang and Yang 1994) comb. n. (= *Nixe (Nixe) mitificus* Kang and Yang 1994 =*Paracinygmula mitifica* Bauernfeind and Soldán 2012;

*Rhithrogeniella obscura* (Kang and Yang 1994) comb. n. (= *Nixe (Nixe) obscurus* Kang and Yang 1994 =*Paracinygmula obscura* Bauernfeind and Soldán 2012.

*Nixe/Paracinygmula* is therefore restricted to the Holarctic Realm, whereas *Rhithrogeniella* is Oriental, reported from Taiwan, continental Southeast Asia and from Java and Sumatra in the Sunda Islands. The genus is presently recorded neither from Borneo (Braasch 2011; Sartori et al. 2003) nor from the Philippines (Braasch 2011).

Based on the Bayesian majority-rule consensus tree reconstructed from the combined data set in Vuataz et al. (2013), *Rhithrogeniella* appears more related to the tribe Compsoneuriini sensu Sartori (2014) *(Compsoneuria, Compsoneuriella* and *Notonurus)*, than to other Ecdyonurinae *(Thalerosphyrus, Asionurus, Atopopus, Afronurus)*, although low posterior probability and bootstrap support does not allow to determine its exact relationships. It is possible that further studies may show that a new tribe should be established to accommodate this genus.

One remaining question concerns the presence or absence of titillators on *Rhithrogeniella* male genitalia. These structures are mentioned by Soldán and Braasch (1986) in the male subimago of *Rhithrogeniella tonkinensis* as well as in the subimago of *Rhithrogeniella ornata* (Soldán and Braasch 1986, page 204). Although we have not dissected the holotype (the only male imago of *Rhithrogeniella* known at the moment), we feel confident that this specimen lacks median titillators. The structures of the subimago male genitalia, illustrated in Fig. 4, are not “well-developed, cylindrical medial titillators with sclerotized apices” (Soldán and Braasch 1986), because they are only cuticular processes, weakly sclerotized except at the apex which is spine-like. In all Ecdyonurinae subimagos which do possess true titillators, these structures are deeply sclerotized, profoundly rooted inside the penis lobes, and are present in the imaginal stage after the subimaginal molt. The cuticular processes mentioned in *Rhithrogeniella* are thus likely to disappear with the subimaginal molt. We conclude therefore that, to our present knowledge, *Rhithrogeniella* lacks true titillators. The supposed male imago of *Rhithrogeniella tonkinensis* briefly described by Braasch (1990) possesses median titillators as well as a very curious styliger fig, with two large triangular processes. These processes should already be present in the subimago and easily visible; but because they are present neither in the male subimago of *Rhithrogeniella ornata* nor *Rhithrogeniella tonkinensis*, we can conclude that the male of Braasch (1990) is misassociated and possibly belongs to a species of *Afronurus*.

## Differential diagnosis

*Rhithrogeniella ornata* appears to be closely related to *Rhithrogeniella tonkinensis*, known from Vietnam and Thailand. It differs from the latter mainly by the ornamentation of the crown of the galea-lacinia, with 13–14 comb-shape setae, median ones with 10–11 teeth, whereas *Rhithrogeniella tonkinensis* bears only 10–11 comb-shape setae, median ones with 6–8 teeth. Additional nymphal characters, and egg chorionic structure are also very similar. Differences between subimagos of both species proposed by Soldán and Braasch (1986) are tenuous, and rely mainly on the tarsal composition of the hind leg (1=2=5>3≥4 in *Rhithrogeniella ornata* compared to 1=5>2=3>4 in *Rhithrogeniella tonkinensis*). Tibia of foreleg is distinctly shorter than the femur in *Rhithrogeniella ornata*, whereas it is reported as subequal to the femur in *Rhithrogeniella tonkinensis*. Subimaginal male genitalia are rather similar, although penis lobes appear more rounded in *Rhithrogeniella ornata* than in *Rhithrogeniella tonkinensis*.

Compared to the Taiwan species, *Rhithrogeniella ornata* can be easily separated from *Rhithrogeniella littoralis* and *Rhithrogeniella obscura* by the shape of the mandibles with inner and outer incisors subequal in length (inner incisor much shorter in *Rhithrogeniella littoralis* and *Rhithrogeniella obscura*), from *Rhithrogeniella mitifica* and *Rhithrogeniella obscura*, by the higher number of teeth on the comb-shape setae of the galea-lacinia (4–5 teeth only in *Rhithrogeniella mitifica* and *Rhithrogeniella obscura* vs 10–11 in *Rhithrogeniella ornata*), from *Rhithrogeniella mitifica* by the shape of the spines on the posterior margin of the tergites (pointed in *Rhithrogeniella ornata* vs tabular in *Rhithrogeniella mitifica*), and from *Rhithrogeniella littoralis* by the much more elongated gill VII.

## Supplementary Material

XML Treatment for Rhithrogeniella ornata
